# Stretching the Rules: Monocentric Chromosomes with Multiple Centromere Domains

**DOI:** 10.1371/journal.pgen.1002777

**Published:** 2012-06-21

**Authors:** Pavel Neumann, Alice Navrátilová, Elizabeth Schroeder-Reiter, Andrea Koblížková, Veronika Steinbauerová, Eva Chocholová, Petr Novák, Gerhard Wanner, Jiří Macas

**Affiliations:** 1 Biology Centre of the Academy of Sciences of the Czech Republic, Institute of Plant Molecular Biology, České Budějovice, Czech Republic; 2 Ultrastructural Research, Biozentrum der LMU München, Planegg-Martinsried, Germany; Duke University, United States of America

## Abstract

The centromere is a functional chromosome domain that is essential for faithful chromosome segregation during cell division and that can be reliably identified by the presence of the centromere-specific histone H3 variant CenH3. In monocentric chromosomes, the centromere is characterized by a single CenH3-containing region within a morphologically distinct primary constriction. This region usually spans up to a few Mbp composed mainly of centromere-specific satellite DNA common to all chromosomes of a given species. In holocentric chromosomes, there is no primary constriction; the centromere is composed of many CenH3 loci distributed along the entire length of a chromosome. Using correlative fluorescence light microscopy and high-resolution electron microscopy, we show that pea (*Pisum sativum*) chromosomes exhibit remarkably long primary constrictions that contain 3–5 explicit CenH3-containing regions, a novelty in centromere organization. In addition, we estimate that the size of the chromosome segment delimited by two outermost domains varies between 69 Mbp and 107 Mbp, several factors larger than any known centromere length. These domains are almost entirely composed of repetitive DNA sequences belonging to 13 distinct families of satellite DNA and one family of centromeric retrotransposons, all of which are unevenly distributed among pea chromosomes. We present the centromeres of *Pisum* as novel “meta-polycentric” functional domains. Our results demonstrate that the organization and DNA composition of functional centromere domains can be far more complex than previously thought, do not require single repetitive elements, and do not require single centromere domains in order to segregate properly. Based on these findings, we propose *Pisum* as a useful model for investigation of centromere architecture and the still poorly understood role of repetitive DNA in centromere evolution, determination, and function.

## Author Summary

During cell division, DNA packed in chromosomes must be perfectly distributed between daughter cells. Centromeres play a crucial role in this process. Current centromere biology maintains that stable chromosomes can be either monocentric, with one functional domain located at a single position, or polycentric, with multiple domains located along the entire chromosome. We found that pea chromosomes are different, exhibiting very large single centromeres containing multiple functional domains, thus representing a novel intermediate type of centromere. We demonstrate that all of the functional centromere domains in the pea are tightly associated with clusters of distinct satellite DNA families, indicating their role in centromere evolution. Our results support the idea that the tandem organization of repeating units, but not their primary sequences, is an important co-determinant of functional centromere domains.

## Introduction

Centromeres are chromosome domains that are essential for faithful chromosome segregation during cell division. It is maintained that stable chromosomes can be either monocentric, possessing centromere activity within a cytologically distinguishable primary constriction, or polycentric (holocentric), lacking a primary constriction and exhibiting centromere activity over nearly the entire chromosome length. It is assumed that polycentric chromosomes have arisen multiple times during evolution because they are present in independent eukaryotic lineages. However, the mechanism of transition from monocentric to polycentric chromosomes is not yet known, nor has any intermediate between the two types been documented.

A functional centromere domain is currently defined as a chromosomal region upon which a kinetochore assembles and to which microtubules of the mitotic spindle are attached [1]. One of the fundamental inner kinetochore proteins is a centromere-specific histone H3 variant, referred to as CenH3 (CenpA in animals, CID in *Drosophila melanogaster*, Cse4 in *Sacharomyces cerevisiae* or HCP3 in *Caenorhabditis elegans*) [2]. Contrary to canonical histone H3, which is extremely conserved in all eukaryotes, CenH3 shows considerable variability between species [2]. Since it is present in the functional centromere of all eukaryotes studied so far, it has become a universal marker of centromeric chromatin. It has been shown that functional centromere domains of monocentric chromosomes are composed of small intermingling subunits, 10 to 50 Kbp in length, containing nucleosomes with either CenH3 or canonical H3 histones [3], [4]. During chromosome condensation, it is postulated that the CenH3-containing subunits accumulate toward the poleward face of the centromere and are the foundation for a single compact kinetochore complex [4]–[6]. While the CenH3-containing chromatin forms a single domain localized within primary constriction of the monocentric chromosomes, it is distributed as contiguous loci in a linear axis over nearly the entire length of the polycentric ones [7]–[9].

In our previous work we found that chromosomes of the pea (*Pisum sativum*) exhibit unusually long primary constrictions containing multiple clusters of distinct families of satellite DNA [10]. This contrasts with most species investigated so far, that exhibit short primary constrictions and often a single family of satellite DNA that is common to all centromeres of a given karyotype. Although the role of satellite DNA for centromere function is not yet fully understood, the centromere domains tend to be established upon its arrays [11]. In order to uncover how the size and DNA sequence composition of primary constrictions are related to the organization of the functional centromere domains in pea chromosomes, we employed molecular and cytological techniques combined with next-generation sequencing of genomic and ChIP-enriched DNA followed by bioinformatics analysis. We demonstrate that the elongated constrictions in pea chromosomes exhibit 3–5 explicit CenH3-containing regions, a centromeric novelty. We introduce this as a meta-polycentric organization, representing the first example of an intermediate between monocentric and polycentric centromeres. We show that these domains are tightly associated with clusters of 13 distinct families of satellite DNA, indicating an important role of satellite DNA in centromere function and evolution.

## Results/Discussion

**Figure 1:**
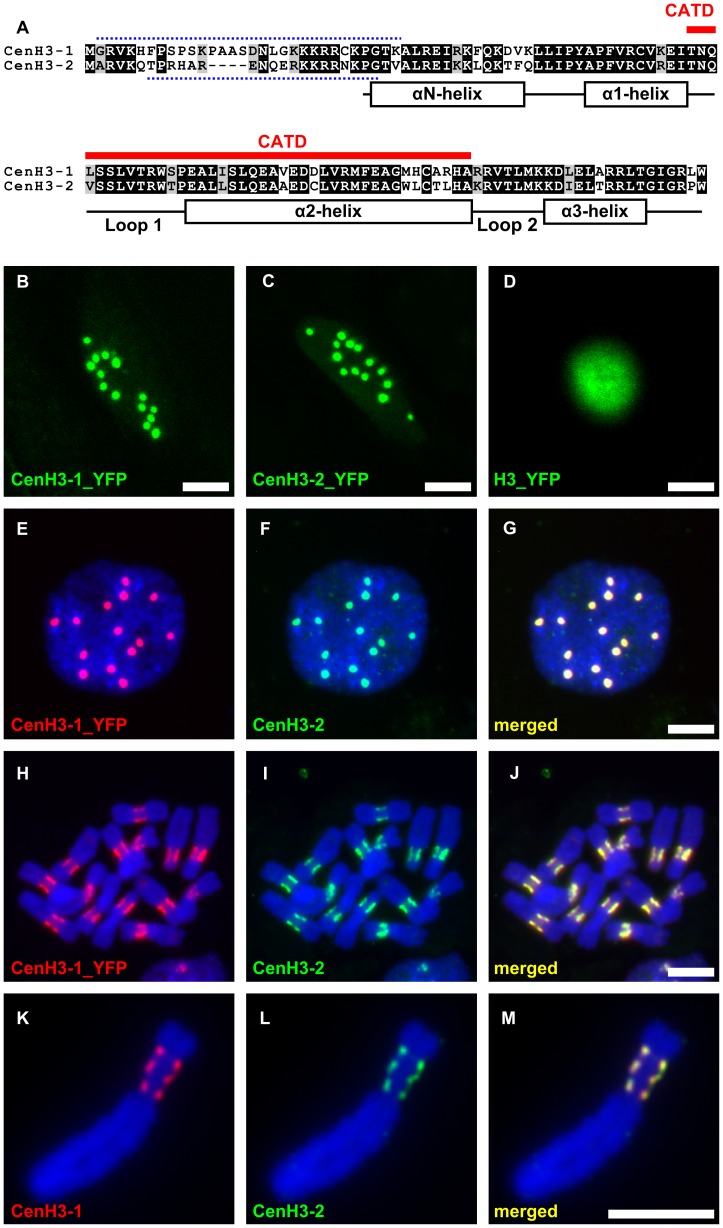
Pea has two variants of the CenH3 that fully colocalize in centromeres of all chromosomes. A: Alignment of protein sequences of the pea CenH3 histones. Red line above the alignment marks a putative centromere targeting domain (CATD). Dotted lines above and below the alignment show the peptide sequences which were used as antigens to produce antibody to CenH3-1 and CenH3-2, respectively. Secondary structure of the histone fold domain is depicted below the alignment. B–C: Direct visualization of fusion proteins of CenH3-1 or CenH3-2 with YFP revealed 14 foci in the interphase nucleus, corresponding to the number of chromosomes in diploid cells. D: Fusion protein of canonical H3 with YFP is localized in whole nucleus. ELISA assays of the two CenH3 antibodies revealed low level of cross-reaction of the CenH3-1 antibody to the peptide designed from the CenH3-2 (data not shown). As we could not determine if the cross-reactivity was sufficient to produce signal after detection *in situ*, the colocalization experiments were performed using highly-specific antibodies to YFP and CenH3-2 in hairy root lines expressing CenH3-1_YFP. E–J: Detection of CenH3-1_YFP (red) and CenH3-2 (green) revealed full colocalization of the two proteins both in interphase nucleus (E–G) and metaphase chromosomes (H–J). K–M: Fully overlapping signals were observed also using simultaneous detection of CenH3 proteins with antibodies to CenH3-1 (red) and CenH3-2 (green) as shown on the example of chromosome 3 possessing three distinct domains containing CenH3. This indicates that either of the two antibodies was capable of detecting all functional centromere domains and that the gaps between individual domains lack CenH3 of any type. Bar = 5 µm.

**Figure 2:**
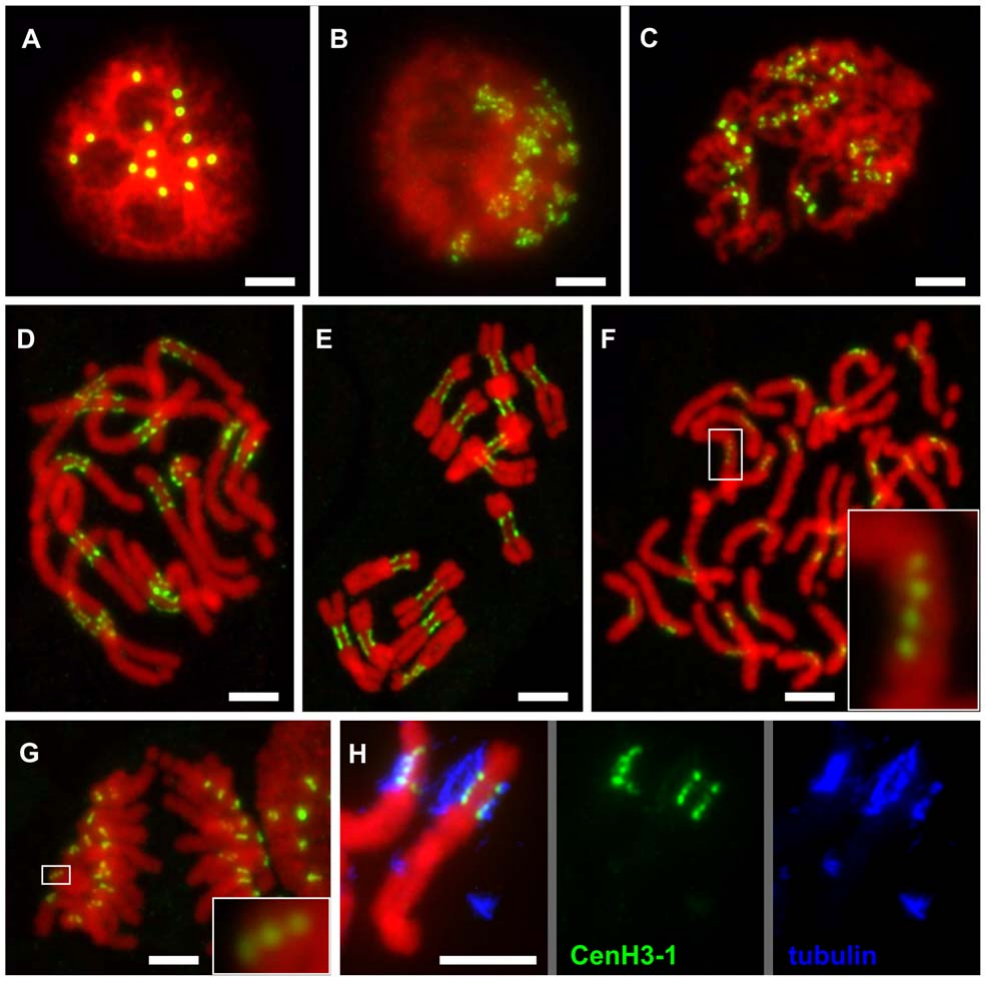
Organization of CenH3-containing domains during the cell cycle. A: Number of CenH3-containing domains in interphase nucleus corresponds to the number of chromosomes in diploid cells (2n = 14). B–D: Mitotic chromosomes at the early prophase (B), prophase (C) and prometaphase (D) show multiple domains containing the CenH3 which are clearly separated with chromatin segments lacking the CenH3. E–G: The separation of CenH3-containing domains becomes less apparent with the progress of chromatin condensation: metaphase (E), spread of single-chromatid anaphase chromosomes (F), anaphase (G). However, the multiple domain structure can be observed in some cases even in the highly condensed anaphase chromosomes (detail windows in F and G). H: Simultaneous detection of CenH3-1 (green) and tubulin (blue) revealed that microtubules are attached to all CenH3 containing domains as shown for chromosome 3. All chromosomes were counterstained with DAPI (pseudocolored in red). Bar = 5 µm.

**Figure 3:**
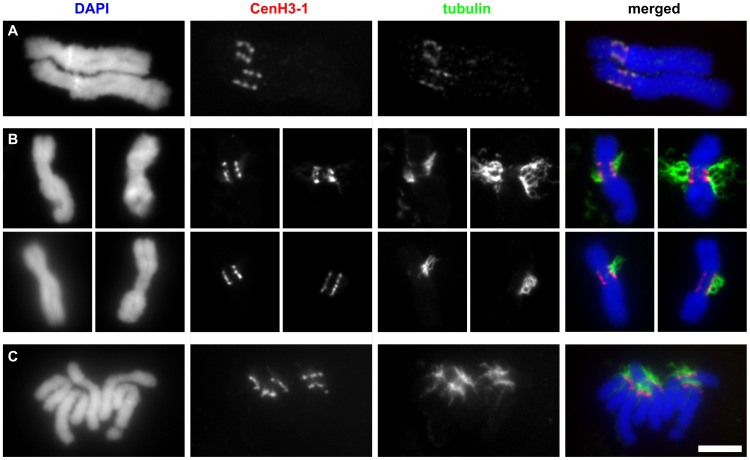
The CenH3-containing domains are fully colocalized with tubulin. A: Immunodetection of tubulin and CenH3-1 on two isolated metaphase chromosomes 3. Although isolated chromosomes never remained attached to microtubules they rarely exhibited weak tubulin signals which fully colocalized with CenH3-1. B–C: Detection of tubulin and CenH3-1 on chromosomes prepared using squash technique. The squash technique allowed some chromosomes to remain attached to microtubules. Whenever present, the remnants of mitotic spindle attached to chromosomes at all CenH3-containing domains on both metaphase (B) and anaphase (C) chromosomes. Bar = 5 µm.

Although CenH3 is, with few known exceptions [12]–[15], encoded by a single gene in diploid species [16], in the pea genome we identified two divergent copies, designated as *CenH3-1* and *CenH3-2* (GenBank accession numbers JF739989 and JF739990), sharing only 55% identity. CenH3-1 and CenH3-2 proteins differ both in length and sequence, being composed of 123 and 119 amino acid residues, respectively, and having 72% identity (Figure 1A). Transformation experiments using pea hairy-root tissue cultures expressing constructs containing cDNA fragment coding either of the CenH3 variants fused with yellow fluorescence protein (YFP) gene demonstrated that, despite the sequence differences, both CenH3 variants target all 14 centromeres in diploid nuclei (Figure 1B–1C). Immunodetection experiments further showed that both CenH3 variants completely co-localized in interphase and mitotic chromosomes (Figure 1E–1M). In sharp contrast to other investigated species, including *Vicia faba* [17], [18] which is a close relative of the pea, we found that primary constrictions of all pea chromosomes contain not a single but multiple functional centromere domains (Figure 2). These domains were best distinguished in prophase and prometaphase chromosomes exhibiting three to five domains that are clearly separated by chromatin blocks lacking CenH3. With increasing condensation in late metaphase and anaphase, the domains came very close to one another or merged into a single extended layer at the poleward face of the primary constriction. However, even in fully condensed chromosomes, the CenH3 within the layer was not evenly distributed, showing intermingling fluorescent signal spots of varying intensity in a row, resembling a string of beads (Figure 2F–2G). The chromosome segments delimited by the two most distant domains were roughly estimated to represent 9.5–18.8% of individual chromosomal DNA which corresponds to 69–107 Mbp (Table S1). To verify that all CenH3-containing domains are indeed sites of kinetochore formation, we carried out the simultaneous immunodetection of CenH3 and tubulin, a protein of the mitotic spindle which is also localized to the kinetochore [19]. Tubulin signals detected in the kinetochore colocalized with those of CenH3, indicating that all CenH3-containing regions are truly functional centromere domains (Figure 2H and Figure 3).

**Figure 4:**
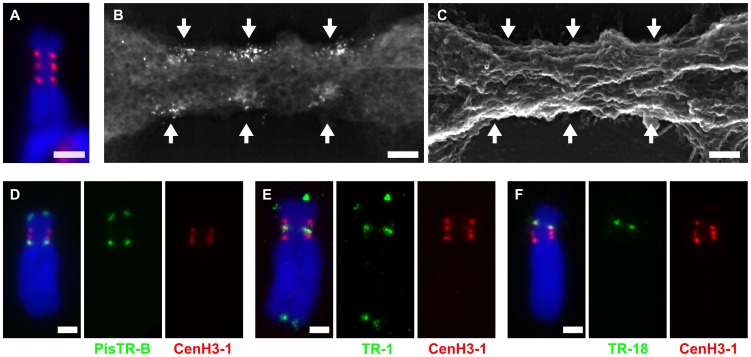
Organization and DNA sequence composition of CenH3-containing domains in chromosome 3. A–B: Primary constriction of chromosome 3 contains three functional centromere domains as defined by the presence of CenH3-1. Correlative fluorescence and scanning electron microscopy images of the same chromosome using FluoroNanogold showed that the three domains recognized with fluorescence (A, red signals) are composed of multiple foci from markers (bright spots) near the surface of the primary constriction (B, backscattered electron micrograph). C: Secondary electron micrograph image of the same chromosome. The primary constriction exhibits few chromomeres and a typical longitudinal orientation of fibrillar substructures, to which the CenH3 domains roughly correspond. The arrows mark the CenH3-1 containing regions. D–F: Detection of three different families of satellite DNA by FISH (green) combined with immunodection of CenH3-1 (red). Each of the functional centromere domains is composed of different family of satellite DNA; the domain closest to the long arm is composed of PisTR-B (D), the middle one of TR-1 (E) and the one closest to the short arm of TR-18 (F). Chromosomes were counterstained with DAPI (blue). Bar = 2 µm (A and D–F) or 0.2 µm (B–C).

We selected chromosome 3 for the investigation of the centromere structure and the CenH3 distribution with higher resolution using correlative light fluorescence microscopy (LFM) and field emission scanning electron microscopy (FESEM). In this approach, CenH3-containing regions were detected with FluoroNanogold, allowing for the investigation of the same chromosomes with both techniques (Figure 4A–4C). Three distinct and strongly labeled regions, located on either side of the longitudinal chromosome axis, were detected using both LFM and FESEM imaging (Figure 4A–4B). Secondary electron (SE) imaging of the primary constriction revealed longitudinally oriented fibrillar structures interspersed with chromomeres in the range of about 200 nm in diameter (Figure 4C). Backscattered electron (BSE) detection of CenH3 markers showed that labeled regions are composed of discrete multiple signals (Ø 10–15 nm) from markers near the surface, as well as diffuse regions from markers in the interior of the centromere (Figure 4B). Subsequent investigation with a dual beam focused ion beam (FIB) and FESEM system allowed direct visualization of CenH3 markers in the centromere interior (Video S1). Measured by 5 nm milling steps, markers occurred between 10 nm and approx. 200 nm from both poleward centromere surfaces (Figure S1). High resolution 3-D reconstruction of the CenH3 distribution revealed that very few of the CenH3 signals actually occur at the chromosome surface (Video S2), indicative of other kinetochore factors at the chromatin-microtubule interface.

**Figure 5:**
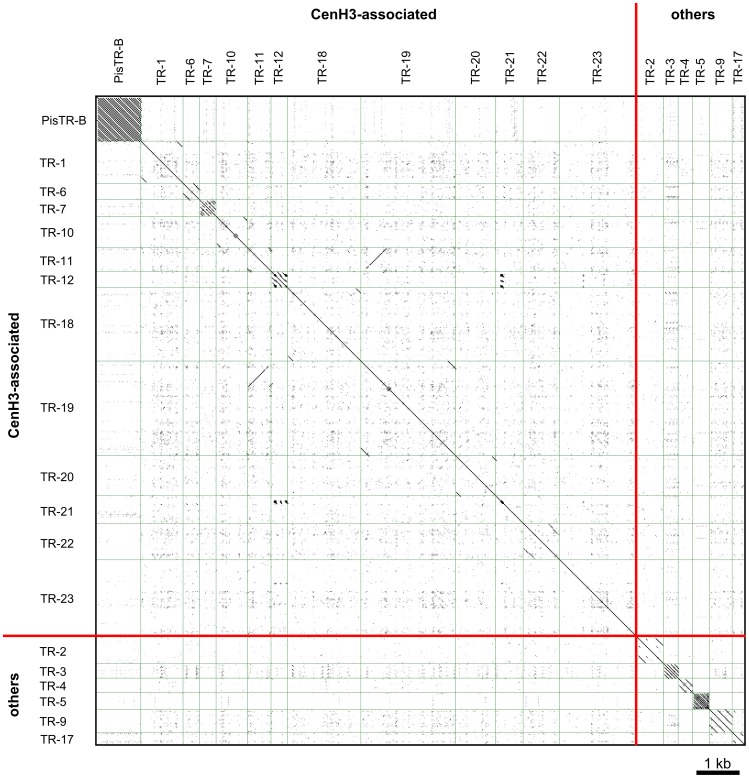
All to all dot-plot comparison of the pea satellite repeats. With exception of TR-11 and TR-19, the sequences of different satellite DNA families show no similarity. A fragment of long monomer of TR-19 shows high similarity to TR-11. FISH experiments revealed that all loci of TR-19 repeat contain also TR-11, but only some of TR-11 loci contain TR-19 (Figure 6 and data not shown), indicating that these two repeats should be considered as different families. Sequences used for dot-plot comparison are provided in Dataset S1.

**Figure 6:**
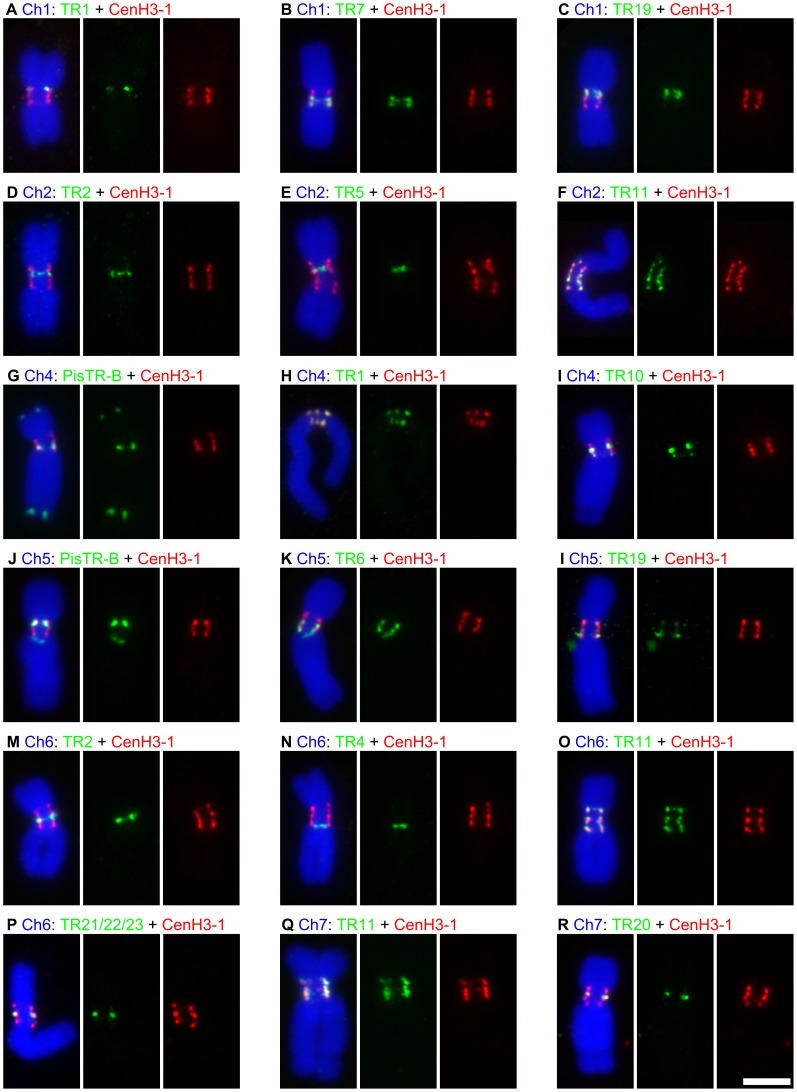
Association of satellite DNA sequences with CenH3-containing domains. The figure shows results of simultaneous detection of different families of satellite DNA by FISH (green) and immunodection of CenH3-1 (red). A–C, F–L and O–R: All families of satellite DNA showing high ChiP enrichment were found to be colocalized with regions containing CenH3-1. D–E and M–N: The DNA families with no ChIP enrichment but present in primary constrictions were indeed found outside of the CenH3-containing domains. Colocalization of CenH3-1 and satellite DNA families on chromosome 3 is shown in the Figure 4. Bar = 5 µm.

In order to uncover DNA sequence composition of the functional centromere domains, we carried out chromatin immunoprecipitation sequencing (ChIP-seq) which produced approximately 9.5 and 19.7 million 35 nt long reads for ChIP and its input control sample, respectively. As the whole pea genome sequence is not yet available, we employed as a reference sequences that were obtained by paired-end Illumina sequencing of the pea nuclear DNA at 0.48× coverage (20.5 million reads, 100 nt in length; all deep sequencing data related to this study have been deposited into the Sequence Read Archive under the study accession number ERA079142 (http://www.ebi.ac.uk/ena/data/view/ERA079142)). Sequences associated with CenH3 were identified based on the ratio between ChIP and input sequences mapped either to sequence clusters representing the most abundant repeats of the pea genome or to each reference read (Figure S2). The latter approach revealed a total of 354 717 reference reads (1.73%) showing at least 10-fold enrichment which were grouped into sequence clusters based on their mutual similarity, as described previously [20]. Further analysis of the clustered sequence data revealed that a vast majority (99%) of the ChIP-enriched sequences belongs to 13 distinct families of satellite DNA (Table 1) and one family of Ty3/gypsy retrotransposon belonging to the CRM clade of chromoviruses [21]. This data suggests that functional centromere domains are established almost exclusively upon repetitive DNA sequences. These repeats differed considerably from one another, not only in their primary sequences but also in the size of repeating units and abundance in genome (Table 1, Figure 5 and Dataset S1). The association of all these repeats with functional centromere domains was confirmed using fluorescence *in situ* hybridization (FISH) combined with immunodetection of CenH3-1 (Figure 4D–4F and Figure 6) which allowed the assignment of each CenH3-containing domain to some of the identified satellites. These experiments also showed that only the repeats with a high ChIP/input ratio are specific to functional centromere domains, while those with lower ChIP/input ratio (e.g. PisTR-B and TR-12) are localized predominantly outside of these domains (Table 1, Figure 6 and data not shown). In addition to the ChIP-enriched satellites, we included in these experiments three families of satellite DNA (TR-2, 4, and 5) that are known to occupy primary constrictions but showing no ChIP enrichment (ChIP/input<1.1), which indeed localized outside of CenH3-containing regions (Figure 6D–6E and 6M–6N). Contrary to most other species that possess a relatively high level of sequence homogenization among all centromeres [22] DNA sequence composition of the centromere domains in the pea varied between chromosomes as well as between individual domains of the same chromosome. The only exception was chromosome 2 that contains a single centromeric satellite family (TR-11, Figure 6F).

**Table 1:** Characterization of satellite DNA families identified in the pea.

Family^1^	ChIP enrichment^2^	Genome proportion (%/Mbp)^3^	Monomer size^4^	AT content	Localization on chromosomes^5^						
					1	2	3	4	5	6	7
PisTR-B	20.5	1.374/59.09 ^a^	50	0.72	T	T	C	C, T	C,P	T	T
TR-1	51.7	0.021/0.91 ^b^	867	0.67	C	-	C, T	C	-	-	-
TR-6	59.3	0.011/0.49 ^b^	245	0.76	-	-	-	-	C	-	-
TR-7	49.7	0.135/5.82 ^b^	164	0.73	C	-	-	-	-	-	-
TR-10	76.3	0.01/0.43 ^a^	659	0.74	-	-	-	C, P	-	-	-
TR-11	74.9	0.103/4.44 ^b^	510	0.76	C	C	-	-	C	C	C
TR-12	5.4	0.006/0.29 ^a^	120	0.69	I	P, I	I	C, I	I	I	I
TR-18	82.5	0.013/0.57 ^b^	1644	0.74	-	-	C	-	-	-	-
TR-19	65.9	0.033/1.43 ^b^	2094	0.77	C	-	-	-	C	-	-
TR-20	50.7	0.004/0.19 ^b^	867	0.76	-	-	-	-	-	-	C
TR-21	44.0	0.006/0.24 ^b^	642	0.73	-	-	-	-	-	C	-
TR-22	102.9	0.004/0.17 ^b^	881	0.76	-	-	-	-	-	C	-
TR-23	10.7	0.002/0.09 ^b^	1813	0.69	-	-	-	-	-	C	-
TR-2	0.6	0.207/8.92 ^a^	440	0.68	-	P	-	-	P	P	-
TR-3	0.3	0.058/2.48 ^a^	82	0.79	-	-	P	I	-	-	-
TR-4	0.6	0.200/8.61 ^a^	172	0.67	-	-	-	-	-	P	-
TR-5	1.1	0.151/6.51 ^a^	54	0.65	-	P	-	-	-	-	-
TR-9	0.4	0.009/0.37 ^a^	189	0.79	T	T	-	-	-	-	T
TR-17	0.5	0.012/0.52 ^a^	191	0.74	-	-	-	-	-	-	I

1 Satellite DNA families which are not at all associated with CenH3 are included in the bottom part of the table, starting from TR-2.

2 ChIP enrichment values were calculated using the top one thousand most repetitive clusters build from 2 million randomly selected reads (Figure S2A). The only exceptions included TR21 and TR-22 which were not represented within this data set. Their enrichment values were therefore calculated from clusters build from ChIP-enriched reads.

3 Genome proportion was estimated as a proportion of reads belonging to a given repeat family to the total number of analyzed reads. The table shows the greater estimate of those calculated from either the top one thousand most repetitive clusters (a) or clusters build from all ChIP-enriched reads (b). The total size of the repeats was calculated from the genome proportion assuming that the genome size of the pea is 4 300 Mbp.

4 Monomer sizes were estimated based on contigs assembled from clustered reads and confirmed by sequencing PCR products amplified from genomic DNA.

5 C – centromeric (associated with CenH3), P – pericentromeric (located in or adjacent to primary constriction but not associated with CenH3), I – intercalary, T – (sub-)telomeric.

It has already been shown that functional centromere domains of monocentric chromosomes are composed of intermingling subunits, 10 to 50 Kbp in length, containing nucleosomes with either CenH3 or canonical H3 histones [3], [4]. Although it is not yet well understood how the centromeric chromatin folds during chromosome condensation in mitosis, all current models postulate that CenH3-containing subunits are brought together toward the poleward face of the centromere to form a single compact kinetochore [4]–[6]. As the size of the subunits is relatively small, they can be observed only at the finest resolution of chromatin fiber but not at the level of condensed mitotic chromosomes. Thus, none of the current models allow for large intermingling domains at the poleward side of mitotic chromosomes, as are observed in the pea. On the other hand, the high resolution 3-D distribution of CenH3 in individual centromere domains (Video S2) resembles that postulated for single centromere domains of previously investigated centromeres [6].

From a molecular point of view, therefore, the pea chromosomes have multiple centromere domains, yet they have only one primary constriction at metaphase. Chromosomes with two or more functional centromeres are usually unstable due to the formation of anaphase bridges leading to chromosome breakage. One exception is when the two centromeres are physically so close that they are able to fuse into a single centromere without disturbing mitosis [23]. The maximum distance between two centromeres that still allows faithful segregation of dicentric chromosomes was estimated to be about 20 Mbp [24]. Taking into account the size of the chromosome segments delimited by the two outermost functional centromere domains (Table S1) and the total number of these domains in individual chromosomes, the distance between any two domains is likely to be either below this limit or not exceed it considerably. This probably allows the multiple domains to act in concert, assuring that pea chromosomes are stable during mitosis, behaving as functional monocentrics.

The high diversity of DNA sequence composition of functional centromere domains observed in the pea is unprecedented, but it concurs with the notion that centromeres are determined rather epigenetically (for review see [25]). On the other hand, similarly to most other species investigated thus far [11], all of the centromere domains in the pea are made up of satellite DNA, indicating that the tandem organization of repeating units co-determines centromere domains. This converges with the recently proposed role of repetitive DNA in centromere function relying on a formation of covalently closed DNA loops made by inter-repeat homologous recombination [26]. However, the tandem arrangement of the repeating units is clearly not the only precondition for a DNA sequence to function as a centromere because some clusters of satellite DNA located within the primary constrictions are not associated with CenH3.

The structure of large pea centromeres is reminiscent of holocentric, also called polycentric, chromosomes that exhibit numerous discrete centromere domains extending over nearly the entire length of the chromosome [7]. As with the pea, the centromere domains congregate during mitosis to form a composite, linear-like kinetochore [7], [9], [27]. The sizes of segments of pea chromosomes delimited by the outermost functional centromere domains (Table S1) approach or even exceed the size of entire polycentric chromosomes of some species, including *C. elegans* (14–21 Mbp) and *Luzula nivea* (155 Mbp on average) [28], [29]. A portion of centromere domains in *Luzula nivea* is composed of scattered clusters of satellite LCS1 [30], suggesting that satellite DNA is an important centromere determinant in at least some holocentric chromosomes. Remarkably, the LCS1 satellite has a similarity to the RCS2 (CentO) which is the major centromeric satellite of monocentric chromosomes of some Oryza species [31].

Although the mechanism of transition from monocentric chromosomes to polycentric ones is not yet known and may differ between organisms, a conceivable scenario for *Luzula nivea* could be that it occurred as a consequence of spreading of centromere-competent satellite(s). If this is the case, then pea chromosomes with multiple distinct clusters of CenH3-associated satellites might represent an intermediate “meta-polycentric” type between monocentric and polycentric chromosomes. However, it has been postulated that centromere expansion causes deleterious effects which in turn create pressure for its suppression, possibly by changes in key factors such as CenH3 or Cenp-C [32]. This explains why the centromere expansion is not an infinite process and why the size of centromeres of most eukaryotic species remains limited to relatively small chromosome domains. Therefore, we assume that pea centromeres are more likely to be or to have already been suppressed in their expansion rather than continue their spreading further into noncentromeric regions. It is tempting to speculate that the presence of two *CenH3* genes in the pea is somehow related to the unusual centromere structure. However, it is impossible to conclude from the available data whether the ancient duplication of *CenH3* genes and their diversification occurred before or after the centromere expansion. Further research is necessary to fully understand the cause and effects of these unusual features of pea centromeres. Establishing the pea as a new model organism for centromere investigation will contribute to a better understanding of centromere chromatin organization and dynamics during the cell cycle as well as the still elusive role of repetitive DNA in centromere evolution, determination and function.

## Materials and Methods

### Plant material

All experiments were performed using pea (*Pisum sativum*) cultivar Carrera. Seeds were obtained from Osiva Boršov (Boršov nad Vltavou, Czech Republic).

### Identification and cloning *CenH3* genes in the pea

Search for *CenH3* gene was done using sequence data obtained from next-generation sequencing of the pea transcriptomes of roots, leaves and flowers (about 72.2 million 50 nt long reads generated by SOLiD sequencing, unpublished data) and the pea genome at the depth corresponding to 0.48× coverage (about 20.5 million 100 nt long reads generated by Illumina sequencing). It revealed two different variants of *CenH3* genes, designated as *CenH3-1* and *CenH3-2*. Long fragments of the *CenH3* genes were amplified using PCR. *CenH3-1* was amplified with primers PN_ID317 (AAA AGC GAA ATT GAA AAT CAA AAT CTG) and PN_ID320 (GAC TCA TTT TAA ATT CTC ATT CTC ATT CTC ATT) while the *CenH3-2* was amplified with primers PN_ID321 (AGT CGC TCT CTG TGT ACA CAA ACT TAA AG) and PN_ID324 (GTT CCA AGA ATT TTA CTT TCC AGA TAG ATA CTT A). Each 30 µl PCR contained 1× PCR buffer, 0.2 mM dNTP, 0.3 µM of each primer, 2% (w/v) DMSO, 0.3 U LA DNA polymerase (Top-Bio, Prague, Czech Republic) and 150 ng pea genomic DNA. The reaction profile consisted of a denaturation step (94°C/60 s) followed by 35 cycles of 94°C/15 s, 61°C/30 s and 68°C/3 min. Amplified fragments were cloned into the pCR4-TOPO plasmid vector (Invitrogen, Carlsbad, CA). Consensus sequences derived from sequencing of three randomly selected clones of each *CenH3* variant have been deposited in GenBank under accession numbers JF739989 and JF739990.

The coding regions of the *CenH3-1*, *CenH3-2*, and canonical *H3* genes were obtained by RT-PCR amplification. Total RNA was isolated either from leaves of the pea (*CenH3-1* and *CenH3-2*) or Medicago truncatula (*H3*) using Trizol reagent (Invitrogen, Carlsbad, CA) and treated with DNase I (Ambion, Austin, TX). First strand synthesis was achieved with a SuperScript III First-Strand Synthesis System for RT–PCR kit (Invitrogen, Carlsbad, CA), following the manufacturer's recommendations and employing random hexamers as primers. A sample of 5 ng of the resulting cDNA was used as template for a 25 µl PCR containing 1× PCR buffer, 0.2 mM dNTP, 0.2 µM of each primer (PN_ID76: ATG GGT AGA GTT AAG CAC TTC C and PN_ID69: CCA AAG TCT TCC TAT TCC TGT AAG for *CenH3-1*, PN_ID313: ATG GCG AGA GTT AAA CAA ACA and PN_ID314: CCA AGG TCT TCC TAT CCC G for *CenH3-2*, PN_ID93: ATG GCA CGT ACC AAG CAA ACT G and PN_ID95: AGC GCG CTC ACC ACG GAT for *H3*), 1.5 mM MgCl_2_ and 1 U Platinum Taq polymerase (Invitrogen, Carlsbad, CA). The amplification regime consisted of an initial denaturation step (94°C/3 min), followed by 35 cycles of 94°C/30 s, 55°C/50 s and 72°C/60 s and a final extension of 72°C/10 min.

### Fusion constructs and transformation

RT-PCR amplified fragments encoding for CenH3 and H3 histones were cloned into the pCR8/GW/TOPO entry vectors using pCR8/GW/TOPO TA Cloning Kit (Invitrogen, Carlsbad, CA). The fragments in appropriate orientation were subsequently recombined into destination vector pEarleyGate104 (obtained from TAIR; http://www.arabidopsis.org/), allowing for C-terminal fusion with YFP. The recombination reaction was carried out using Gateway LR Clonase II Enzyme Mix (Invitrogen, Carlsbad, CA) according to the manufacturer's instructions. Nucleotide sequences of all constructs were verified by sequencing.

Transgenic hairy root cultures expressing the reporter gene were obtained by transformation of *P. sativum* plants by *Agrobacterium tumefaciens* C58C1 carrying both hairy root inducing plasmid pRiA4 and pEarleyGate104 vector possessing either of the constructs. The transformation was performed by injecting *Agrobacterium* suspension into stems of 7-days-old seedlings cultivated *in vitro* on 50% Murashige and Skoog medium (Duchefa, Haarlem, Netherlands). The seedlings were grown at 20°C (16 h photoperiod). After 3–4 weeks of cultivation, hairy roots emerging from the inoculation sites were excised and placed on solid Gamborg B5 medium (Duchefa, Haarlem, Netherlands) supplemented with ticarcillin (500 mg/l) and cefotaxime (200 mg/l) for elimination of bacteria, and glufosinate ammonium (10 mg/l) for selection of lines carrying the YFP constructs. Hairy root cultures were grown in Petri dishes at 24°C in the dark and transferred to fresh B5 medium once a month.

The images of transgenic cells expressing the constructs of CenH3 and H3 (both YFP at C-terminal end) were captured using confocal microscope Olympus FV1000 and processed in FW10-ASW software.

### Chromatin immunoprecipitation (ChIP)

Nuclei were isolated from 10 g of young leaves as described previously [10]. The isolated nuclei were centrifuged at 400 g for 5 min at 4°C and resuspended in 3 ml micrococcal nuclease (MNase) buffer (10% sucrose, 50 mM Tris-HCl pH 7.5, 4 mM MgCl_2_, 1 mM CaCl_2_). The chromatin suitable for ChIP was prepared by digestion of the nuclei with MNase (150 units of the enzyme per 3 ml of nuclei) for 40–60 min at 37°C. The reaction was stopped by adding 0.5 M EDTA to a final concentration of 20 mM and samples were centrifuged at 13,000 g for 5 min at 4°C. The supernatant containing well digested chromatin was saved (fraction 1) while the pellet containing poorly digested chromatin was resuspended in 200 µl MNase buffer and redigested with 15 units of MNase for 5 min at 37°C. The reaction was stopped with EDTA and centrifuged as described above. The supernatant was mixed with the fraction 1 and a 200 µl aliquot was taken from the chromatin sample for DNA isolation to serve as an input control sample. The rest of the mixture was diluted with the same volume of ChIP incubation buffer (50 mM NaCl, 20 mM Tris-HCl pH 7.5, 5 mM EDTA, 0.2 mM phenylmethylsulfonyl fluoride, 1× protease inhibitor cocktail (Sigma-Aldrich, St. Louis, MO)). ChIP was done using Immunoprecipitation Kit – Dynabeads Protein G (Invitrogen, Carlsbad, CA) according to manufacturer's instructions with some modifications. The ChIP was preceded with a precleaning step; 2 ml of the chromatin were mixed with Dynabeads Protein G from 50 µl of the stock, incubated on a rotator for 4 h at 4°C, and finally separated from the beads using a magnet. Antibody binding was done for 2 h at 4°C in 200 µl of Ab binding and washing buffer containing magnetic beads from 50 µl and 30 µg of the antibody to CenH3-1. The beads with bound antibody were mixed with the precleaned chromatin and the mixture was incubated with rotation overnight at 4°C. Immunoprecipitated complexes were washed 4×5 min using 200 µl of the washing buffer. Elution of the chromatin was done using 2×100 µl of preheated elution buffer (1% sodium dodecyl sulfate, 0.1 M NaHCO_3_) for 30 min at 65°C. DNA from the ChIP and input control samples was isolated using ChIP DNA Clean and Concentrator Kit (Zymo Research, Irvine, CA). Sequencing of the input and ChIP DNA was done using Illumina technology producing 36 nt long reads (Creative Genomics, Shirley, NY).

### Chromosome preparations, immunodetection, and fluorescence *in situ* hybridization (FISH)

Most immunostaining and FISH experiments were done using chromosomes isolated from root tip meristem cells synchronized using 1.25 mM hydroxyurea and blocked at metaphase using 15 µM oryzalin or 10 µM APM as described previously [33]. The squash preparations were made in 1× phosphate-buffered saline (PBS) buffer by squashing synchronized root tip meristems fixed in 4% formaldehyde for 25 min and digested with 2% cellulase and 2% pectinase in 1× PBS for 85 min at 28°C. To avoid potential influence of the synchronization on signal patterns of CenH3, we employed also squash preparations made of nonsynchronized meristems which produced the same results. Affinity purified polyclonal antibodies to peptides designed from CenH3-1 (GRV KHF PSP SKP AAS DNL GKK KRR CKP GTK C) and CenH3-2 (TPR HAR ENQ ERK KRR NKP GC) histones were custom-produced (Genscript, Piscataway, NJ) in rabbit and chicken, respectively. Commercially available antibodies included rabbit antibody to GFP (Invitrogen, Carlsbad, CA; catalog number A11122) and mouse antibody to α-tubulin (Sigma-Aldrich, St. Louis, MO; catalog number T6199). Prior to incubation with either antibody, the slides were incubated in PBS-T buffer (1× PBS, 0,1% Tween 20, pH 7,4) for 30 min at room temperature (RT). The slides were incubated with primary antibodies diluted in PBS-T overnight at 4°C. Dilution ratios were as follows: 1∶1000–5000 for both CenH3 antibodies, 1∶500 for YFP antibody, and 1∶50 for antibody to α-tubulin. Following two washes in 1× PBS for 5 min, the antibodies were detected by anti-rabbit-Rhodamine Red-X-AffiniPure (1∶500, Jackson ImmunoResearch, Suffolk, UK; catalog number 111-295-144), anti-chicken-DyLight488 (1∶500, Jackson ImmunoResearch; catalog number 103-485-155), anti-mouse-FITC (1∶100, Abcam, Cambridge, UK; catalog number ab6785) or anti-rabbit-Alexa488-NanoGold (Nanoprobes, Yaphank, NY) in PBS-T buffer for 1 h at RT. After final washes of PBS, the slides were counterstained with 4′,6-diamino-2-phenylindole (DAPI) and mounted in Vectashield mounting medium (Vector Laboratories, Burlingame, CA). In double immunodetection experiments, the two primary or secondary antibodies were incubated together and appropriate control experiments were performed to exclude non-specific binding.

For a combined detection of the CenH3 proteins and the satellite repeats, the immunodetection procedure was followed by FISH. After CenH3-1 detection and washing, the slides were immediately postfixed in 4% formaldehyde in 1× PBS for 10 min at RT, and dehydrated in series of 70% and 96% ethanol, 5 min at RT each. Chromosome denaturation was carried out in a PCR buffer (Promega, Madison, WI) supplemented with 4 mM MgCl_2_ for 2 min at 94°C. The preparation of hybridization probes, hybridization conditions, and probe detection were set up as described by Macas *et al.* [10]. The chromosomes were examined using a Nikon Eclipse 600 microscope. Images were captured with a DS-Qi1Mc cooled camera and analyzed by NIS Elements 3.0 software (Laboratory Imaging, Praha, Czech Republic).

The chromosome sizes in Mbp were estimated from relative chromosome lengths of individual chromosomes and haploid genome size of 4 300 Mbp [34] using following formula: genome size×relative chromosome length/100. The relative chromosome lengths were taken from Neumann *et al.* [35]. Centromere size was estimated using chromosomes stained with DNA-binding fluorescent dye DAPI as a proportion of integrated fluorescence density within the segments delimited by the two outermost CenH3-containing regions compared to that of whole chromosome. The measurements were done on mitotic chromosomes at prometaphase to metaphase.

### Identification of ChIP–enriched sequences

Reads from ChIP and input sequencing were trimmed at both 5′ and 3′ end, leaving 31 bp sequences which were further subjected to quality filtering (reads containing more than one base with a quality lower than 20 were removed). This left 9,515,830 and 19,699,136 high quality reads in the ChIP and input data sets, respectively. In order to get a suitable reference needed for the identification of ChIP-enriched sequences, we sequenced the pea nuclear genome at about 0.48× coverage (20,527,392 reads 100 bp in length) using Illumina technology producing pair end reads. All sequence data has been deposited to the Sequence Read Archive under the study accession number ERA079142 (http://www.ebi.ac.uk/ena/data/view/ERA079142). The mapping of the ChIP and input reads to the reference sequences was done using PatMaN program [36], allowing for up to two differences between the query and the hit, both of which could be indels with a maximum total size of four bases. The ChIP enrichment was calculated as a ratio between a proportion of ChIP and input reads mapped to a reference. This was done using two approaches. The first approach employed clusters calculated due to computational limitations from only 2 million randomly selected reference sequences as described previously [20]. The clusters grouped together reads derived from individual repeat families or their fragments. Top 1000 clusters with the highest genome representation were used to determine their ChIP-enrichment. In this approach the ChIP and input reads were mapped to the reference sequences present in the clusters. ChIP or input sequence read was assigned to a given cluster if it had a hit to at least one reference read from that cluster. It should be noted that each read could be assigned to only a single cluster. Reads with equal similarity to reference sequences from more than one cluster were assigned to the one with a higher genome representation. The ChIP enrichment values were calculated from the total number of reads assigned to individual clusters. Advantage of this approach was that it allowed to determine ChIP-enrichment values for all major repeat families present in the pea genome. On the other hand, it missed all single and low-copy sequences.

The other approach relied on determination of the ChIP enrichment values for each of 20,527,392 reference sequences. Only those showing the ChIP enrichment of at least 10 were selected to build the clusters as described in Novák *et al.* [20]. Thus, these clusters were made only of sequences putatively derived from CenH3-associated regions, providing about 10-fold deeper coverage as compared to the clusters build from 2,000,000 reads. In addition, this analysis involved all available sequences regardless of their repetitiveness.

Type of ChIP-enriched repeat families was determined by their similarity to previously characterized repeats [10] and by their graph shape [20]. Tandem arrangement of novel satellite repeat families was confirmed by PCR using primers directed outwards from a putative monomer instead towards each other (data not shown). In such PCR design, a product of expected size can be obtained only if monomer sequences have head-to-tail tandem organization. The only non satellite centromere repeat, the Ty3/gypsy retrotransposon belonging to the CRM clade, was determined by high level of similarity to a full-length element described recently [21].

### Field emission scanning electron microscopy (FESEM)

Prior to FESEM, immunolabeled (Alexa488-NanoGold, see above) specimens were washed in 100% ethanol to remove mounting medium, washed in distilled water, and silver enhanced for 4 min according to the manufacturer's instructions (HQ Silver, Nanoprobes, Yaphank, NY). After washing again in distilled water, specimens were dehydrated in acetone, critical point dried from CO_2_, cut to size and mounted onto aluminum stubs. Specimens were carbon-coated by evaporation (Balzers high vacuum evaporator BAE 121, Liechtenstein) to a layer of 3–5 nm for orthogonal (top-view) FESEM and examined at 10–30 kV with an Hitachi S-4100 field emission scanning electron microscope equipped with a Everhard-Thornby chamber secondary electron (SE) detector and a YAG-type back-scattered electron (BSE) detector (Autrata). SE and BSE images were recorded simultaneously with DigiScan hardware and processed with Digital Micrograph 3.4.4 software (both Gatan, Pleasanton, CA).

Dual beam focused ion beam/field emission scanning electron microscopy (FIB/FESEM) investigations were performed on a Zeiss Auriga CrossBeam Workstation, a field emission scanning electron microscope equipped with a Gallium ion beam, in-lens, chamber SE and EsB detectors (Carl Zeiss, Germany) as described in Schroeder-Reiter *et al.* [37]. For FIB/FESEM sectioning the specimens were carbon-coated to 10 nm for stability. Milling steps were defined at 5 nm. The electron beam voltage was 1 kV; the EsB grid was set at 900 V. In the cut-and-view mode FESEM images were recorded using a ratio of 70% BSE to 30% SE signal detection. Specimens were tilted to an angle of 54°; image recordings were tilt-compensated.

Marker molecules were quantified by counting the number of signal spots per milled centromere section. Animation of FIB milling and partial alignment functions were performed with ImageJ (Rasband, W.S., ImageJ, U. S. National Institutes of Health, Bethesda, MD, http://rsb.info.nih.gov/ij/). Segmentation and labeling of signals and chromatin, 3D reconstructions, and animations were achieved using Amira software (Visage Imaging, Richmond, Australia).

## Supporting Information

Dataset S1 Set of sequences representing different satellite DNA families in the pea.
(FASTA)

Figure S1 Diagram showing the number of CenH3 markers per centromere domain of chromosome 3. The markers were counted in milled sections of the pea chromosome 3 shown in the Figure 4A. Individual markers (approx. 10 nm in diameter) could be counted in sequential high resolution FIB/FESEM micrographs (a series of 126 milled sections at a milling thickness of 10 nm per section). 3D reconstruction of labeled centromere region (box) designates the three separate centromere domains for which the CenH3 marker count is shown in the diagram. For all three domains, the marker number peaks around mid-chromatid, with very few markers near the poleward centromere surfaces (arrowheads) and a minimum at the central axis of the chromosome (dotted line) over approximately 200 nm.
(TIF)

Figure S2 Plots of ChIP enrichments. Sequences associated with the CenH3 were identified using two approaches. A: Using sequence data from the top 1000 clusters calculated from 2 million randomly selected reference reads. Each cluster represents a group of reads belonging to the same repetitive element or its fragment. Chip enrichment was calculated as a proportion between ChIP and input reads mapped to each of the clusters. Note that vast majority of clusters is ChIP-depleted indicating that most repetitive sequences are localized outside of centromeric region. This computationally less demanding approach was sufficient to identify all major centromeric repeats but failed to find the less abundant ones. B–C: Using all 20.5 million reference reads. B: Scatter plot showing number of ChiP and input reads mapped to the reference reads. C: Histogram of ChIP enrichment values. Red and green lines mark the enrichment values of 10 and 1, respectively. A total of 354 717 reads showing at least 10-fold enrichment were used for clustering which allowed to identify and characterize additional, less abundant, centromeric repeats including mainly TR-21, TR-22, and TR-23 (Table 1).
(TIF)

Table S1 Size estimation of regions delimited by the most distant CenH3-containing domains.
(DOC)

Video S1 Animation of a FIB/FESEM series of SEM micrographs (1 kV, signal ratio 30% SE and 70% BSE) showing sequential images of milled surface revealing the exposed centromere interior. The series is of 124 images acquired after sequential 5 nm milling steps.
(MOV)

Video S2 An animated rotation around a 3D reconstruction of the centromere of pea chromosome 3 showing CenH3 distribution (CenH3 markers = yellow) together with total chromatin distribution (chromatin = transparent magenta), showing clearly that the majority of CenH3 markers are located toward the poleward centromere boundaries; only few CenH3 markers are localized on the lateral centromere surface. The three strongly labeled regions are composed of numerous individual signals; few diffuse signals are also found between and bordering these concentrated regions.
(MPG)
