# Supplementary figures and images for: Stretching the Rules: Monocentric Chromosomes with Multiple Centromere Domains
Source: PLoS Genet. 2012 Jun 21;8(6):e1002777. doi: 10.1371/journal.pgen.1002777 (PMC3380829; doi:10.1371/journal.pgen.1002777)

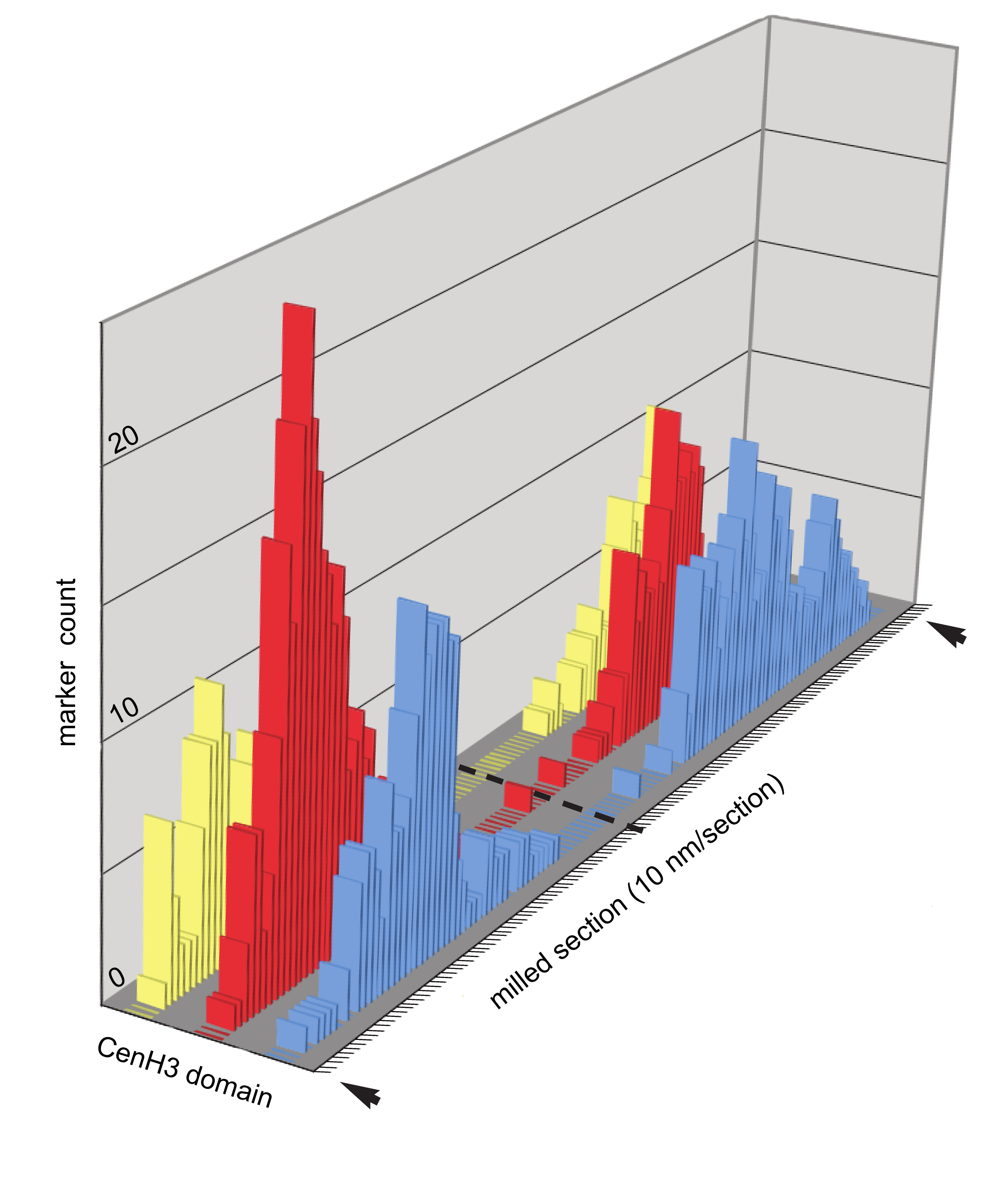

Supplement: Figure S1 — Diagram showing the number of CenH3 markers per centromere domain of chromosome 3. The markers were counted in milled sections of the pea chromosome 3 shown in the Figure 4A. Individual markers (approx. 10 nm in diameter) could be counted in sequential high resolution FIB/FESEM micrographs (a series of 126 milled sections at a milling thickness of 10 nm per section). 3D reconstruction of labeled centromere region (box) designates the three separate centromere domains for which the CenH3 marker count is shown in the diagram. For all three domains, the marker number peaks around mid-chromatid, with very few markers near the poleward centromere surfaces (arrowheads) and a minimum at the central axis of the chromosome (dotted line) over approximately 200 nm. (TIF) [file pgen.1002777.s002.tif]

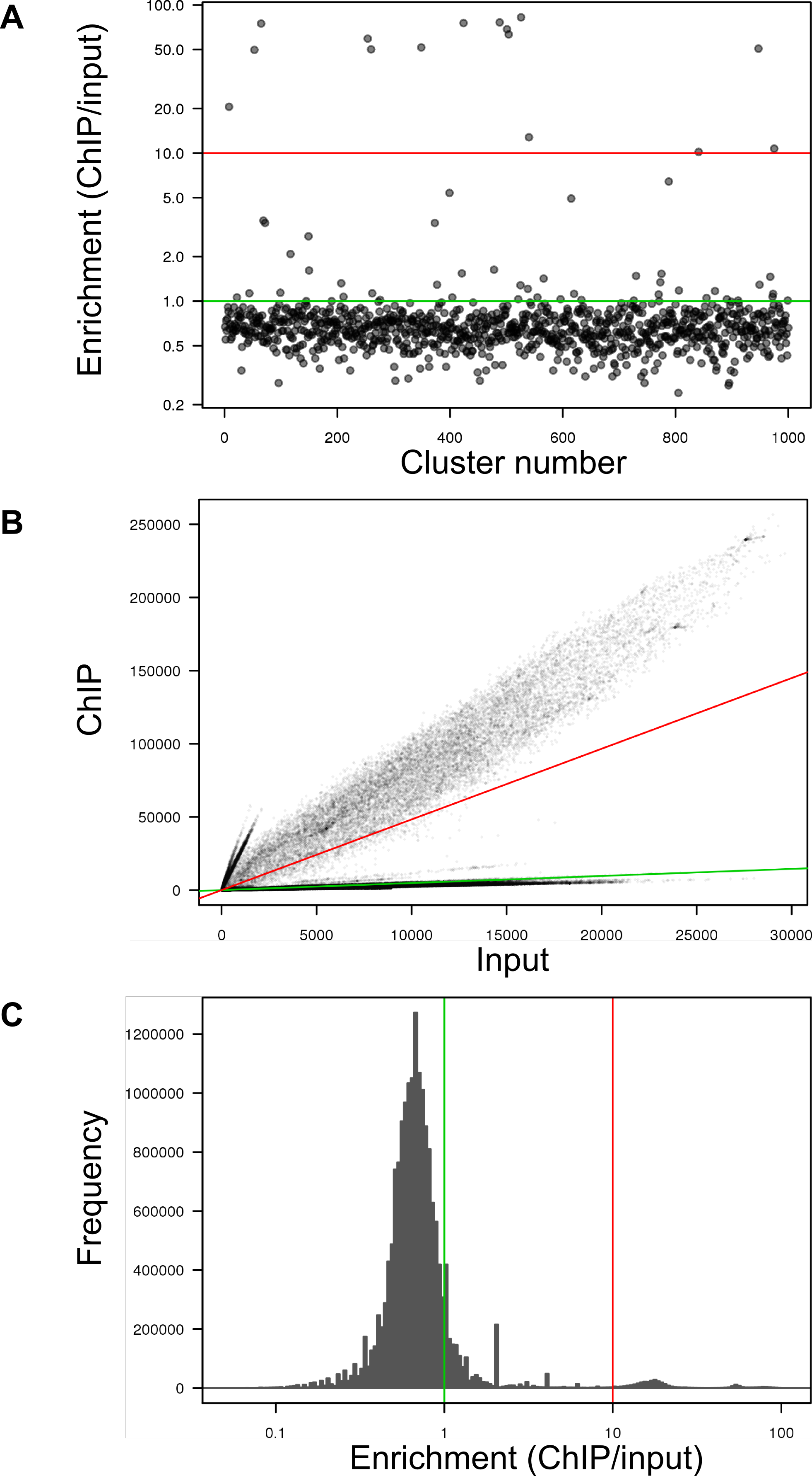

Supplement: Figure S2 — Plots of ChIP enrichments. Sequences associated with the CenH3 were identified using two approaches. A: Using sequence data from the top 1000 clusters calculated from 2 million randomly selected reference reads. Each cluster represents a group of reads belonging to the same repetitive element or its fragment. Chip enrichment was calculated as a proportion between ChIP and input reads mapped to each of the clusters. Note that vast majority of clusters is ChIP-depleted indicating that most repetitive sequences are localized outside of centromeric region. This computationally less demanding approach was sufficient to identify all major centromeric repeats but failed to find the less abundant ones. B–C: Using all 20.5 million reference reads. B: Scatter plot showing number of ChiP and input reads mapped to the reference reads. C: Histogram of ChIP enrichment values. Red and green lines mark the enrichment values of 10 and 1, respectively. A total of 354 717 reads showing at least 10-fold enrichment were used for clustering which allowed to identify and characterize additional, less abundant, centromeric repeats including mainly TR-21, TR-22, and TR-23 (Table 1). (TIF) [file pgen.1002777.s003.tif]
