# Supplementary material for: Stretching the Rules: Monocentric Chromosomes with Multiple Centromere Domains
Source: PLoS Genet. 2012 Jun 21;8(6):e1002777. doi: 10.1371/journal.pgen.1002777 (PMC3380829; doi:10.1371/journal.pgen.1002777)
Supplement: Table S1 — Size estimation of regions delimited by the most distant CenH3-containing domains. (DOC) [file pgen.1002777.s004.doc]

**Table S1.** **Size estimation of regions delimited by the most distant CenH3-containing domains.**

| Chromosome | Relative chromosome length [%] / estimated chromosome size [Mbp] ^1^ | Centromere proportion [% / Mbp] ^2^ | STD [% / Mbp] ^3^ |
| --- | --- | --- | --- |
| 1 | 12.65 / 543.95 | 14.3 / 77.7 | 2.1 / 11.3 |
| 2 | 12.04 / 517.72 | 18.8 / 97.3 | 1.5 / 7.6 |
| 3 | 14.06 / 604.58 | 12.9 / 77.9 | 0.8 / 5.0 |
| 4 | 14.39 / 618.77 | 11.9 / 73.5 | 0.9 / 5.8 |
| 5 | 16.86 / 724.98 | 9.5 / 69.1 | 1.4 / 10.3 |
| 6 | 14.39 / 618.77 | 17.4 / 107.4 | 1.3 / 7.8 |
| 7 | 15.68 / 674.24 | 12.7 / 85.5 | 1.4 / 9.3 |

^1^ The chromosome sizes were estimated from relative chromosome lengths of individual chromosomes and haploid genome size of 4 300 Mbp.

^2^ Centromere proportion was estimated based on the proportion of integrated fluorescence density of DAPI-stain within the segments delimited by the two outermost CenH3-containing regions compared to that of whole chromosome.

^3^ Standard deviation (STD) was calculated from the measurements of at least ten chromosomes.
